# A risk-based model for predicting the impact of using condoms on the spread of sexually transmitted infections

**DOI:** 10.1016/j.idm.2017.02.004

**Published:** 2017-03-01

**Authors:** Asma Azizi, Karen Ríos-Soto, Anuj Mubayi, James M. Hyman

**Affiliations:** a Department of Mathematics, Tulane University, New Orleans, LA, 70118, United States; b Department of Mathematical Sciences, University of Puerto Rico, Mayaguez, PR, 00610, United States; c School of Human Evolution and Social Change, Arizona State University, Tempe, AZ, United States; d Simon A. Levin Mathematical Computational Modeling Science Center, Arizona State University, Tempe, AZ, United States

**Keywords:** Mathematical modeling, Sexually transmitted infection (STI), Biased (preferential) mixing, Random (proportional) mixing, Condom-use, Risk (number of partners)

## Abstract

We create and analyze a mathematical model to understand the impact of condom-use and sexual behavior on the prevalence and spread of Sexually Transmitted Infections (STIs). STIs remain significant public health challenges globally with a high burden of some Sexually Transmitted Diseases (STDs) in both developed and undeveloped countries. Although condom-use is known to reduce the transmission of STIs, there are a few quantitative population-based studies on the protective role of condom-use in reducing the incidence of STIs. The number of concurrent partners is correlated with their risk of being infectious by an STI such as chlamydia, gonorrhea, or syphilis. We develop a Susceptible-Infectious-Susceptible (SIS) model that stratifies the population based on the number of concurrent partners. The model captures the multi-level heterogeneous mixing through a combination of biased (preferential) and random (proportional) mixing processes between individuals with distinct risk levels, and accounts for differences in condom-use in the low- and high-risk populations. We use sensitivity analysis to assess the relative impact of high-risk people using condom as a prophylactic intervention to reduce their chance of being infectious, or infecting others. The model predicts the STI prevalence as a function of the number of partners of an individual, and quantifies how this distribution of effective partners changes as a function of condom-use. Our results show that when the mixing is random, then increasing the condom-use in the high-risk population is more effective in reducing the prevalence than when many of the partners of high-risk people have high risk. The model quantifies how the risk of being infected increases for people who have more partners, and the need for high-risk people to consistently use condoms to reduce their risk of infection.

## 1 Introduction

There are approximately 19.7 million new Sexually Transmitted Infections (STIs) every year in the United States of America (Satterwhite et al., 2008). More than half of the people in the U.S. will have an STI at some point in their lifetime (Koutsky, 1997). Mathematical models can provide frameworks to understand the underling epidemiology of STI and how they are correlated to the social structure of the infectious population (Del Valle et al., 2005, Del Valle et al., 2007, Hyman and Li, 1997a, Hyman and Li, 1997b). Transmission-based models can help the medical community to understand and to anticipate the spread of diseases in different populations, and help them to evaluate the potential effectiveness of different approaches for bringing an epidemic under control.

We develop and analyze a continuous risk-based transmission model that can be used to understand the spread of STIs in the adolescents and young adult population. The model predicts the impact of people having different number of concurrent partners or using prophylactics, such as condoms, on the rate the infection spreads. These new equations extend our previous two-risk group STI model for the spread of Chlamydia in heterosexual populations (Azizi, Xue, & Hyman, 2016).

The sexually active population is divided into the susceptible population (S), and the infectious population (I). Once a person has recovered from infection, they are again susceptible to infection. That is, the model has an S→I→S (SIS) structure. Using this model, **we study the impact of variations in number of partners, mixing patterns in selecting partners, and condom-use to determine optimal STI prevention policies. We study how the number of partners of an individual, and how often they use condoms, affect the spread of STIs.**

Chlamydia, gonorrhea, syphilis, and chancroid are highly infectious STIs where the number of partners an infectious person has is one of the most important risk factors in spreading the infection. **In our risk-based integro-differential model, the risk is defined based on the number of partners a person has per year.** The distribution of risk behavior for a population, such as the fraction of the population having multiple partners, affects the spread of STIs. Also, the number of partners that their partners have (their partner's risk) affects the spread of an STI and must be accounted for in the model. Our model accounts for a broad range of risk behavior, defined as the number of partners per year, that is captured as a continuous variable.

We use the terms low-risk and high-risk to differentiate between people with only a few partners per year (<3) and those with high number of partners per year (>3). This model could also be used to include separate core high-risk groups, such as sex workers. However, in the young adult population being modeled, sex-workers are not believed to be a major factor in the spread of highly infectious STIs, like chlamydia.

The risk of contracting STI is primarily a function of a person's risk, the probability that a partner is infectious, and the use of prophylactics (e.g. condoms). We use the selective mixing model developed by Busenberg & Castillo-Chavez, (1991) to capture the heterogenous mixing among people with different number of partners. Our model is closely related to the STI models for the spread of the HIV/AIDS in heterosexual networks (Hyman and Stanley, 1988, Hyman and Stanley, 1989) that distribute the population based on their risk, such as the number of partners (Hyman et al., 2001, Hyman et al., 2003, Hyman and Stanley, 1988, Hyman and Stanley, 1989).

Chlamydia and gonorrhea are transmitted when infected semen or vaginal fluids contact mucosal surfaces. Male latex condoms can, if used correctly, block the discharge of semen or protect the male urethra against exposure to vaginal secretions (Centers for Disease Control Prevention & et al., 2002, p. 2007). Condoms can greatly reduce (but not eliminate) the risk of STI, and are the primary strategy for STI prevention in sexually active individuals worldwide (Centers for Disease Control Prevention & et al., 2002, p. 2007; Mann, Stine, & Vessey, 2002). The condom-use parameter is an aggregated measure that accounts the effectiveness of condoms to prevent spread of infection when used appropriately or inappropriately.

The parameters in the model, such as the distribution of risk, are estimated based on recent surveys (Beadnell et al., 2005, Lescano et al., 2006) and from a pilot study of the number of partners for young sexually active people living in New Orleans. We use local sensitivity analysis to identify the relative importance of condom-use and illustrate how this analysis can be used to prioritize individual-level behavioral strategies based on their predicted effectiveness.

## 2 Mathematical model

We assume a closed steady-state population N(r)=S(t,r)+I(t,r) of people with risk $r\in \left[r_{0},r_{\infty }\right]$ is divided into S(t,r), and I(t,r), where S(t,r) (I(t,r)) is the number of susceptible (infectious) people with risk *r* at time t. The susceptible population becomes infectious at the rate *λ* per year, and infectious population recovers with constant rate *γ* to again become susceptible. We assume both susceptible and infectious people leave the population at the migration rate *μ* per year and **that people maintain the same risk** ***r*** **while in the modeled population**.

Our integro-differential equation model for the spread of STIs is

(2.1) $$\begin{array}{l} \frac{\partial S\left(t,r\right)}{\partial t}=\mu \left(N\left(r\right)-S\left(t,r\right)\right)-\lambda \left(t,r\right)S\left(t,r\right)+\gamma I\left(t,r\right)\text{,}\\ \frac{\partial I\left(t,r\right)}{\partial t}=\lambda \left(t,r\right)S\left(t,r\right)-\gamma I\left(t,r\right)-\mu I\left(t,r\right)\text{,}\\ S\left(0,r\right)=S_{0}\left(r\right),\;I\left(0,r\right)=N\left(r\right)-S_{0}\left(r\right)\text{,} \end{array}$$

where initial distributions of the susceptible and infectious population are given at time t=0.

Note that this model does not distinguish between men and women and is appropriate for homosexual STIs or infections when the distribution of risk and infection incidence in men and women is approximately the same. This also requires that the probability of transmitting the infection from an infectious man to a susceptible woman is approximately the same as the probability of transmission from an infectious woman to a susceptible man. This is a reasonable assumption for some STIs, such as chlamydia, syphilis, and gonorrhea. In the absence of symmetry in the transmission parameters or in the risk behavior in men and women, then the model would need to be extended to a two-sex bipartite model.

We model a population of 15–25 year-old sexually active individuals and assume that individuals enter and leave the modeled population only through aging, that is migration rate is defined as $\mu ={\left[\left(24-15\right)\text{years}\right]}^{-1}=1/\left(10\;\mathrm{years}\right)$. We also assume that everyone aging into the population is susceptible to infection, and that people do not change their risk while in the modeled population. To properly account for changes in risk behavior as the population ages, it would require adding an additional variable (age) and is beyond the scope of our simple model. The risk behavior is distributed in a way that number of people with risk *r* decreases as risk *r* increases, that is there are fewer individuals with many partners. We also assume that there is an exponential distribution for the recovery rate of infectious population with an average infection period 1/γ years.

### 2.1 Transmission rate

The force of infection, or transmission rate, λ(t,r), for susceptible person with risk *r* at time *t*, is the rate that susceptible people with risk *r* become infectious through sexual contact. Here a contact is any sexual activity that can transmit the disease between individuals. The mixing among people with different risks determines if a susceptible person with risk *r* can be infectious by someone infectious with risk $r^{\prime }$.

We define λ(t,r) as the integral of the rate of disease transmission at time *t* from each infectious person with risk $r^{\prime }$, $I\left(t,r^{\prime }\right)$, to the susceptible one by

(2.2) $$\lambda \left(t,r\right)=\int_{r_{0}}^{r_{\infty }}\tilde{\lambda }\left(t,r,r^{\prime }\right)dr^{\prime }\text{.}$$

The rate of disease transmission from the infectious persons with risk r' to the susceptible individuals with risk *r*, $\tilde{\lambda }\left(t,r,r^{\prime }\right)$, is defined as the product of three factors:

$$\tilde{\lambda }\left(t,r,r^{\prime }\right)\begin{array}{cccc} = & \left(\begin{array}{c} \text{Number of }r^{\prime }-\text{risk }\\ \text{ partners of susceptible }\\ \text{with risk r},\text{ per year} \end{array}\right) & \left(\begin{array}{c} \text{ Probability of}\\ \text{disease transmission}\\ \text{ per partner} \end{array}\right) & \left(\begin{array}{c} \text{Probability that}\\ \text{partner with risk }r^{\prime }\\ \text{ is infectious} \end{array}\right)\\ = & p\left(r,r^{\prime }\right) & \beta \left(r,r^{\prime }\right) & P_{I}\left(t,r^{\prime }\right)\;\text{,} \end{array}$$

where

• $p\left(r,r^{\prime }\right)$ is the partnership mixing function defined as the number of sexual partners per year that a person with risk *r* has with a person with risk $r^{\prime }$, and
• $\beta \left(r,r^{\prime }\right)$ is the probability of disease transmission per partner to a susceptible person with risk *r* from their infectious partner with risk $r^{\prime }$, and
• $P_{I}\left(t,r^{\prime }\right)$ is the probability that a person of risk $r^{\prime }$ is infectious. Here we assume that there is random mixing among individuals with the same risk, $P_{I}\left(t,r^{\prime }\right)=\frac{I\left(t,r^{\prime }\right)}{N\left(r^{\prime }\right)}$.

### 2.2 Partnership formation

In order to compute p(r,r’), we define a mixing distribution function $\rho \left(r,r^{\prime }\right)$, which captures the mixing between people of different risks and is defined as the fraction $\rho \left(r,r^{\prime }\right)$ of partners of a person with risk *r* who have risk $r^{\prime }$. The distribution function $\rho \left(r,r^{\prime }\right)$ is the expected distribution of partners and is typically estimated based on inaccurate survey data or other assumptions. It cannot be as the actual mixing function $p\left(r,r^{\prime }\right)$ since it usually will not satisfy the balance condition that the total number of people with risk *r* with partners of risk $r^{\prime }$ must equal the total number of people with risk $r^{\prime }$ with partners of risk *r*. The partnership mixing function $p\left(r,r^{\prime }\right)$ must satisfy the balance condition $N\left(r\right)p\left(r,r^{\prime }\right)=N\left(r^{\prime }\right)p\left(r^{\prime },r\right)$ and is defined as a function of $\rho \left(r,r^{\prime }\right)$.

We assume that $\rho \left(r,r^{\prime }\right)$ is a linear combination of randomly selected partners with the random mixing distribution $\rho_{rm}\left(r,r^{\prime }\right)$ and partners based on their preference with the biased mixing distribution $\rho_{bm}\left(r,r^{\prime }\right).$. These mixing distribution functions $\rho_{rm}$ and $\rho_{bm}$ are normalized to have unit integral. Feng, Hill, Curns, & Glasser, (2016) used a similar model to account for multi-level mixing of people within a specified group and among the general population.

#### 2.2.1 Random mixing distribution

When the mixing is random (sometimes called proportional mixing), then individuals with risk *r* do not show any preference for their partners based on risk. The random mixing function for the probability that a person of risk *r* picks a partner with risk $r^{\prime }$ is defined by the ratio of total number of partners for all people with risk $r^{\prime }$, $r^{\prime }N\left(r^{\prime }\right)$, to total number of partnerships, $\int_{r_{0}}^{r_{\infty }}uN\left(u\right)du$. Thus, the random mixing distribution

(2.3) $$\rho_{rm}\left(r,r^{\prime }\right)=\frac{r^{\prime }N\left(r^{\prime }\right)}{\int_{r_{0}}^{r_{\infty }}uN\left(u\right)du}\text{,}$$

is independent of the risk *r* of the person seeking a partnership.

#### 2.2.2 Biased mixing distribution

In our biased (associative or preferential) mixing model, we assume homophily (love of the same) where people with risk *r* prefer to have partners with similar risk. We also assume that people at high risk have partners with a broader range of risk than people at low risk. That is, the standard deviation, σ(r), for the distribution of risk of partners of a person with risk *r* is an increasing function of *r*. This is in agreement with the study by Lescano et al., (2006) that observed the casual and long-term partners of people with many partners are mostly casual partners with few contacts (sexual contacts) per partnership. They also observed that the partners of people with few partners are more often longer term relationships with more contacts per partnership.

**Fig. 1:**
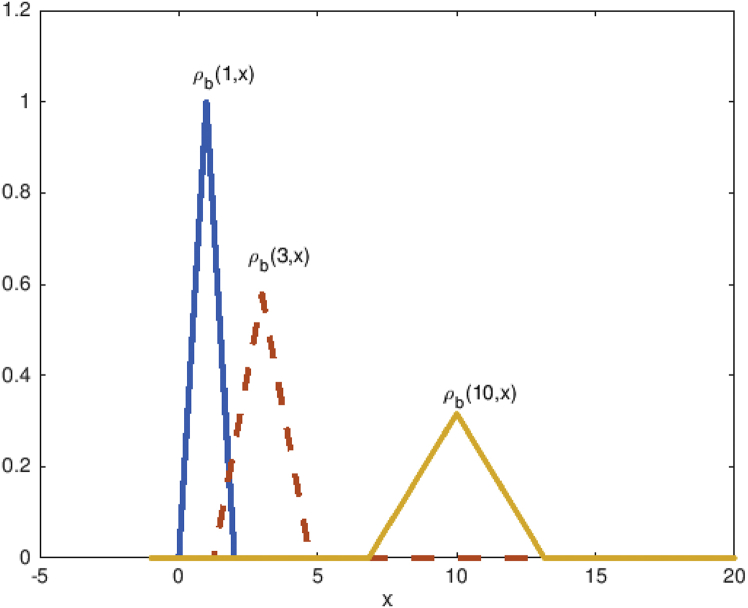
Plot of the triangle (hat) biased mixing function $\rho_{bm}\left(r,x\right)$ for r=1,3,10. As the risk *r* increases, the mixing function becomes fatter and shorter to capture the effect that partners of higher-risk people have a broader range of risk than that of lower-risk people. This is similar to the mixing function used by Hyman & Stanley, (1988).

We define the biased mixing distribution $\rho_{bm}\left(r,r^{\prime }\right).$ for the probability that a person with risk *r* prefers to have a partner with risk $r^{\prime }$ from the range $r^{\prime }\in \left[r-\sigma \left(r\right),r+\sigma \left(r\right)\right]$:

(2.4) $$\rho_{bm}\left(r,r^{\prime }\right)=\left\{ \begin{array}{cc} \frac{-|r^{\prime }-r|+\sigma \left(r\right)}{\sigma {\left(r\right)}^{2}} & |r^{\prime }-r|\leq \sigma \left(r\right)\\ 0 & elsewhere\text{,} \end{array}\right.$$

which satisfies the condition $\int_{-\infty }^{\infty }\rho_{bm}\left(r,r^{\prime }\right)dr^{\prime }=1\text{.}$ Fig. 1 shows how the biased function $\rho_{bm}$ is wider for the higher risk groups.

#### 2.2.3 Combination of random and biased mixing distributions

We assume people choose some of their partners based on their preference (biased mixing) and other partners are chosen randomly from the whole population (random mixing). We define the preference level *ε* as fraction of partners of a person with risk *r* are selected preferentially and the rest are selected randomly, then we can express the mixing distribution as a convex combination of $\rho_{rm}$ and $\rho_{bm}$:

(2.5) $$\rho \left(r,r^{\prime }\right)=\varepsilon \rho_{bm}\left(r,r^{\prime }\right)+\left(1-\varepsilon \right)\rho_{rm}\left(r,r^{\prime }\right)\text{.}$$

When ε=0 the mixing is random, and when ε=1 it is purely biased mixing. Otherwise, a person with risk *r* chooses an *ε* fraction of his/her partners with a hat distribution of people with risk $r^{\prime }\in \left[r-\sigma \left(r\right),r+\sigma \left(r\right)\right]$, and chooses the other partners randomly from all risk value groups.

#### 2.2.4 Partnership mixing function

The partnership function $p\left(r,r^{\prime }\right)$ is the number of partners a person with risk *r* has with someone of risk $r^{\prime }$ per year. A person with risk *r* wants to have $r\rho \left(r,r^{\prime }\right)$ partners with risk $r^{\prime }$, therefore, all individuals with risk *r* want to have $r\rho \left(r,r^{\prime }\right)N\left(r\right)$ partners with risk $r^{\prime }$. On the other hand, all individuals with risk $r^{\prime }$ want to have $r^{\prime }\rho \left(r^{\prime },r\right)N\left(r^{\prime }\right)$ partners with risk *r*. The balance condition states that if people of risk *r* have $P\left(r,r^{\prime }\right)$ partners with risk $r^{\prime }$, then the people with risk $r^{\prime }$ must have $P\left(r^{\prime },r\right)=P\left(r,r^{\prime }\right)$ partners with risk *r*. We define **actual** number of partnership between people with risk *r* and people with risk $r^{\prime }$ as harmonic average of $r\rho \left(r,r^{\prime }\right)N\left(r\right)$ and $r^{\prime }\rho \left(r^{\prime },r\right)N\left(r^{\prime }\right)$:

(2.6) $$P\left(r,r^{\prime }\right)=2\frac{r\rho \left(r,r^{\prime }\right)N\left(r\right)\times r^{\prime }\rho \left(r^{\prime },r\right)N\left(r^{\prime }\right)}{r\rho \left(r,r^{\prime }\right)N\left(r\right)+r^{\prime }\rho \left(r^{\prime },r\right)N\left(r^{\prime }\right)}\text{.}$$

The distribution $P\left(r,r^{\prime }\right)$ is a compromise for the actual number of partnerships between all people with risk *r* and all people with risk $r^{\prime }$. Therefore, the actual number of partners that a person with risk *r* has with people of risk $r^{\prime }$ is

(2.7) $$p\left(r,r^{\prime }\right)=\frac{P\left(r,r^{\prime }\right)}{N\left(r\right)}\text{.}$$

*Remark:* Harmonic average of two values is closer to the smaller one and this compromise weights the decision on forming a sexual partnership towards the person who is less interested to make partnership.

### 2.3 Probability of transmission per partner

The probability per partner, $\beta \left(r,r^{\prime }\right)$, that a susceptible person of risk *r* is infected by an infectious partner of risk $r^{\prime }$ depends upon the number of contacts (sexual acts) between the two risk groups, $A\left(r,r^{\prime }\right)$, and frequency of condom-use in their contacts, $C\left(r,r^{\prime }\right)$.

#### 2.3.1 Sexual contacts per partnership between risk groups

We define $A\left(r,r^{\prime }\right)$ as the total number of sexual contacts per person per year between the a person with risk *r* and a partner with risk $r^{\prime }$. Since there must be the same as the number of sexual contacts between person of risk $r^{\prime }$ with partner of risk *r*, the balance condition, $A\left(r^{\prime },r\right)=A\left(r,r^{\prime }\right)$ must hold.

Suppose a person with risk *r* desires to have, on average, a(r) sexual contacts per partner per year. We assume that a(r) is a decreasing function of *r*:

(2.8) $$a\left(r\right)=\frac{r_{0}}{r}a_{max}\text{,}$$

where $a_{max}$ is the total number of sexual contacts per year.

Since the number of desired sexual contacts per partner for people of risk *r* is not necessarily equal to the number of desired sexual contacts per partnership for people of risk $r^{\prime }$, $a\left(r\right)\neq a\left(r^{\prime }\right)$, there should be a compromise for the sexual contact balance condition to hold. We define the actual number $A\left(r,r^{\prime }\right)$ of sexual contacts per person between the people in risk groups *r* and $r^{\prime }$ as

(2.9) $$A\left(r,r^{\prime }\right)=2\frac{a\left(r\right)a\left(r^{\prime }\right)}{a\left(r\right)+a\left(r^{\prime }\right)}\text{.}$$

Equation (2.9) satisfies the balance condition, and when there is a conflict, the harmonic average results in the actual number of sexual contacts to be closer to the smaller number desired by the two individuals.

#### 2.3.2 Condom-use as a function of risk

We assume that person with risk *r* desires to use a male-latex condom in c(r) fraction of their sexual contacts. We acknowledge that increased condom-use might have an effect on the risk behavior, however, this is not investigated in this work. We assume that higher-risk people are more likely to use condoms than the lower-risk people (Beadnell et al., 2005, Lescano et al., 2006). Therefore, we define c(r) as increasing function of *r*. We observed that the function

(2.10) $$c\left(r\right):=\alpha \left(\frac{r}{c_{0}+r}\right)\text{,}$$

is a good approximation to survey data and interpolates between the case where people have no partners (hence no condom-use), ${\text{lim}}_{r\to 0}c\left(r\right)=c\left(0\right)=0$, and the limit where people have many partners and use-condoms $\alpha ={\text{lim}}_{r\to \infty }c\left(r\right)$ fraction of contacts.

We define the actual fraction of times that a person of risk *r* uses a condom when having sex with a person of risk $r^{\prime }$ as $C\left(r,r^{\prime }\right)=C\left(r^{\prime },r\right)$ which is computed by taking an appropriate average of c(r) and $c\left(r^{\prime }\right)$. The average will depend if the preference (final decision) is closer to the desired condom-use of the person who prefers to use condoms fewer times, or the person who prefers to use condoms more often.

##### 2.3.2.1 Preference to low condom-use

In this case, we assume that a person who is less likely to use condom is more likely to convince the other to not to use condom. We approximate this situation for partners with risk *r* and $r^{\prime }$ to use a condom in

(2.11) $$C_{l}\left(r,r^{\prime }\right)=\frac{2c\left(r\right)c\left(r^{\prime }\right)}{c\left(r\right)+c\left(r^{\prime }\right)}\text{,}$$

fraction of their contacts.

##### 2.3.2.2 Preference to high condom-use

In this case, a person who is more likely to use condom is more probable to convince the other one to use condom. We approximate this situation by taking the harmonic average of the fraction of contacts people do not use condom (1−c(r) and $1-c\left(r^{\prime }\right)$), and therefore, they use condoms in

(2.12) $$C_{h}\left(r,r^{\prime }\right)=1-\frac{2\left(1-c\left(r\right)\right)\left(1-c\left(r^{\prime }\right)\right)}{2-c\left(r\right)-c\left(r^{\prime }\right)}\text{,}$$

fraction of their contacts.

#### 2.3.3 The probability of transmission with condom-use

We define $\beta_{nc}$ and $\beta_{c}$ as the probabilities of transmission per contact for not using and using a condom, and we assume these probabilities are gender-independent, because unlike the heterosexual transmission of HIV/AIDS, the probability of highly infectious STIs (like chlamydia and gonorrhea) transmission from an infected man to a woman is approximately the same as from an infected woman to a man (Althaus et al., 2011, Quinn et al., 1996, Turner et al., 2006). If the condom is 99% effective in preventing the infection from being transmitted, then probability of transmission when using a condom-use is $\beta_{c}=0.01\beta_{nc}$.

To determine the probability of a susceptible person with risk *r* being infected by their infectious partner with risk $r^{\prime }$ depends on the number of contacts, $A\left(r,r^{\prime }\right)$, and how often they use condoms. If someone uses a condom in $C\left(r,r^{\prime }\right)$ fraction of contacts, then they have a total of $C\left(r,r^{\prime }\right)A\left(r,r^{\prime }\right)$ contacts with condoms and $\left(1-C\left(r,r^{\prime }\right)\right)A\left(r,r^{\prime }\right)$ contacts without condom per unit time. The person with risk *r* does not catch infection from their partner during a condom contact with probability ${\left(1-\beta_{c}\right)}^{C\left(r,r^{\prime }\right)A\left(r,r^{\prime }\right)}$, and for when not using a condom this probability is ${\left(1-\beta_{nc}\right)}^{\left(1-C\left(r,r^{\prime }\right)\right)A\left(r,r^{\prime }\right)}$. Combining these, the probability of a susceptible being infected after one contact by infectious partner with risk $r^{\prime }$ is

(2.13) $$\beta \left(r,r^{\prime }\right)=1-{\left(1-\beta_{c}\right)}^{C\left(r,r^{\prime }\right)A\left(r,r^{\prime }\right)}{\left(1-\beta_{nc}\right)}^{\left(1-C\left(r,r^{\prime }\right)\right)A\left(r,r^{\prime }\right)}\text{.}$$

## 3 Parameter estimation

The model parameters (collected in Table 1), the distribution of risk in the population, and the frequency of condom-use were estimated from recent studies on sexual behavior.

**Table 1:** Model parameters: parameter values are chosen for all simulations unless indicated otherwise.

Parameter	Description	Units	Baseline value	Ref
∫N(r)dr	Total Population size	# of People	10,000	Assumed
$a_{max}$	Max number of sexual contacts per year	Contact× Year^−1^	209	Assumed
*γ*	Per capita recovery rate for human from the infectious state to the susceptible state	Year^−1^	1.43	(Kretzschmar, Welte, Van den Hoek, & Postma, 2001)
$\beta_{nc}$	Probability of transmission per no-condom contact	dimensionless	0.11	(Kretzschmar et al., 2001)
$\beta_{c}$	Probability of transmission per condom contact	dimensionless	0.001	Assumed
*μ*	Migration rate	Year^−1^	0.10	Assumed
$r_{0}\left(r_{\infty }\right)$	Minimum(maximum) number of partners per year	People× Year^−1^	1(50)	Assumed
*ε*	Preference Level	dimensionless	0.60	Assumed
*α*	Fraction of contacts condom-used by high-risk people	dimensionless	0.70	Estimated

### 3.1 Population distribution

A sample of 616 people between ages 15−25 years old reside in Orleans Parish were asked about their number of concurrent partners (Kissinger, 2014). This data (in agreement with other recent studies (Colgate, Stanley, Hyman, Layne, & Qualls, 1989)) show that the partner distribution often follows an inverse cubic power law, $N\left(r\right)\propto r^{-3}$, for $r>r_{0}$. The value of N(r) is chosen for the function to agree with the total population size, ∫N(r)dr, being modeled.

### 3.2 Condom-use

The distribution of risk and condom-use were estimated based on surveys for the sexually active adolescents and young adult populations (Beadnell et al., 2005, Lescano et al., 2006, Reece et al., 2010). Reece et al., 2010, CDC studied rates of condom-use among sexually active individuals in the U.S. population and observed that adolescents reported condom-use during 79.1% of the past 10 vaginal intercourse events. Similar studies (CDC) in sexually active high school students in the U.S. reported that during 1991, 46%, during 2003, 63%, and in 2013, 59% of the students used condoms at their most recent sexual intercourse.

Beadnell et al., (2005) surveyed 8−12 th grade students in a large urban northwest school district annually for seven years. They observed that the younger students were more likely to use condoms and also the students with more partners were more likely to use condoms: the students with many partners used condoms, on average, in 68% of their sexual contacts, while the students with few partners used condoms in 49% of their sexual contacts. The condom-use function (Eq. (2.10)) is in close agreement with their observations (Table 2) with parameters the α=0.69 and $c_{0}=1.35$:

(3.1) $$c\left(r\right)=0.69\frac{r}{1.35+r}\text{.}$$

**Table 2:** The average fraction of condom-use by high school students with different risks and different ages, the result of survey conducted in a large urban northwest high school (Beadnell et al., 2005).

15−16 years old		16−17 years old		17−18 years old	
Risk *r*	Fraction of condom-use	Risk *r*	Fraction of condom-use	Risk *r*	Fraction of condom-use
1.2	0.42	1	0.42	1	0.39
5.4	0.58	2	0.42	2	0.38
		7.4	0.60	4.7	0.50

A simple check shows this function is in close agreement with the survey data: c(2)=0.41, c(4.7)=0.50, c(5.4)=0.58, and c(7.4)=0.60.

## 4 Numerical simulations

Since the total population is constant, we scale our results using proportions. We display our numerical simulations in terms of the nondimensional variables defined by dividing each variable by the steady-state zero-infection equilibrium the total population of individuals with the risk *r*, N(r). That is, we present the numerical simulations in terms of the fraction of the population at risk *r*, i.e susceptible $s\left(t,r\right):=\frac{S\left(t,r\right)}{N\left(r\right)}$ and infectious $i\left(t,r\right):=\frac{I\left(t,r\right)}{N\left(r\right)}$. We define $I^{\text{*}}\left(r\right)$ as the number of and $i^{\text{*}}\left(r\right)$ as the fraction of the people that is infectious at the endemic steady state. In the numerical simulations, all the parameters are fixed with the baseline values given in Table 1, unless specifically stated.

### 4.1 Basic reproduction number

The *basic reproduction number* $R_{0}$ is the number of secondary infections created when a newly infectious person is introduced into a population at the disease-free equilibrium. When $R_{0}>1$ and if a small infectious fraction is introduced into the population, then the STI can grow. When the population is distributed as a function of risk, then it is possible to define a basic reproduction number for each value of risk, or a single $R_{0}$ for the entire population based on the dominant eigenvalue of next generation operator. Using a single $R_{0}$ is useful when studying the impact that changes in the biased mixing and condom-use parameters have on the early growth of an epidemic.

We follow Diekmann, Heesterbeek, & Metz, (1990) and define the basic reproduction number as the spectral radius of the next generation operator defined as

(4.1) $$K\left(r\right)=S\left(t,r\right)\int_{r_{0}}^{r_{\infty }}\tau p\left(r,r^{\prime }\right)\beta \left(r,r^{\prime }\right)I\left(t,r^{\prime }\right)dr^{\prime }\text{.}$$

Here τ=1/(μ+γ) is average time that a person is infectious and $\tau p\left(r,r^{\prime }\right)\beta \left(r,r^{\prime }\right)$ is the expected number of people with risk *r* will be infectious by a single infectious person with risk $r^{\prime }$. Thus, the next generation operator, K(r), is number of secondary cases for over all the infectious people with risk $r^{\prime }$, $I\left(t,r^{\prime }\right)$, and is found by integrating over all possible risk groups. That is, K(r) is the number of secondary cases with risk *r* that arises from all the infectious people $I\left(t,r^{\prime }\right)$. The basic reproduction number $R_{0}$ is the dominant eigenvalue of K(r).

We first partition our integro-differential equation model 2.1 into subdomains for different risk groups, $\left[r_{0},r_{\infty }\right]=\overset{n}{\underset{i=1}{\cup }}\left[r_{i-1},r_{i}\right]$, where $r_{n}=r_{\infty }$ and define the populations on for each risk group as $I_{i}\left(t\right)=\int_{r_{i-1}}^{r_{i}}I\left(t,r^{\prime }\right)dr^{\prime }$ and $S_{i}\left(t\right)=\int_{r_{i-1}}^{r_{i}}S\left(t,r\right)dr$. The equations can then be expressed as

$$\frac{dS_{i}\left(t\right)}{dt}=\mu \left(N_{i}-S_{i}\left(t\right)\right)-\lambda_{i}\left(t\right)S_{i}\left(t\right)+\gamma I_{i}\left(t\right)\text{,}$$

$$\frac{dI_{i}\left(t\right)}{dt}=\lambda_{i}\left(t\right)S_{i}\left(t\right)-\gamma I_{i}\left(t\right)-\mu I_{i}\left(t\right)\text{,}$$

where $\lambda_{i}=\sum_{j}\int_{r_{i-1}}^{r_{i}}p\left(r_{i},r_{j}\right)\beta \left(r_{i},r_{j}\right)I_{j}dr_{j,\;\;\;i=1,...,n.}$

We divide the equations by N(r) and approximate the next generation operator K(r) with the *n*-by-*n* next generation matrix K based on assuming the populations are approximately constant within each risk group and that the population is at the zero-infection equilibrium, $s_{i}=1$. The entries of K are defined by

(4.2) $$k_{ij}=\int_{r_{i-1}}^{r_{i}}\tau p\left(r_{i},r_{j}\right)\beta \left(r_{i},r_{j}\right)dr_{j}\text{.}$$

The basic reproduction number $R_{0}$ defined as the dominant eigenvalue of K, is calculated numerically.

**Fig. 2:**
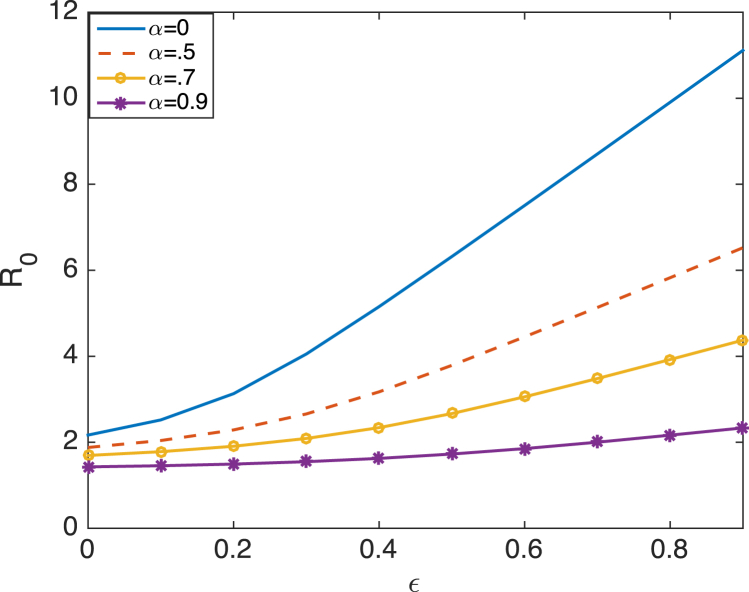
Basic reproduction $R_{0}$ versus preference level *ε* for different condom-uses. The impact of *α* on $R_{0}$ depends on mixing, for more biased mixing *α* has more impact on preventing the infection, however for less biased mixing, the impact of *α* decreases. As *α* decreases $R_{0}$ increases much faster at bigger *ε*s than smaller ones.

Fig. 2 illustrates how $R_{0}$ increases as the amount of biased mixing (*ε*) increases. When a new infection is introduced into the population, if there is even a slight amount of random mixing, someone in the high-risk population will quickly become infected (Hyman & Stanley, 1988). Once this happens, then if the mixing is highly-biased (large *ε*) these infected high-risk people will infect other high-risk people and the epidemic will grow rapidly (large $R_{0}$). If the mixing is close to random mixing (small *ε*), then many of the secondary infections from the early high-risk infected people will have low-risk and the epidemic will grow slower (smaller $R_{0}$). The extreme sensitivity of $R_{0}$ to *α* also is an indication of the importance of educating high-risk individuals in consistent condom-use to prevent infecting others, and the need of the low-risk population in using condoms to protect themselves from infection.

### 4.2 Endemic equilibrium

The fraction of the population that are infectious at the endemic equilibrium infection, $i^{\text{*}}$, depends upon the distribution of risk, N(r), the mixing between people of different risk behaviors, as measured by *ε* in equation (2.5), and the fraction of the contacts condom used by high-risk people, as measured by *α* in equation (2.10).

**Fig. 3:**
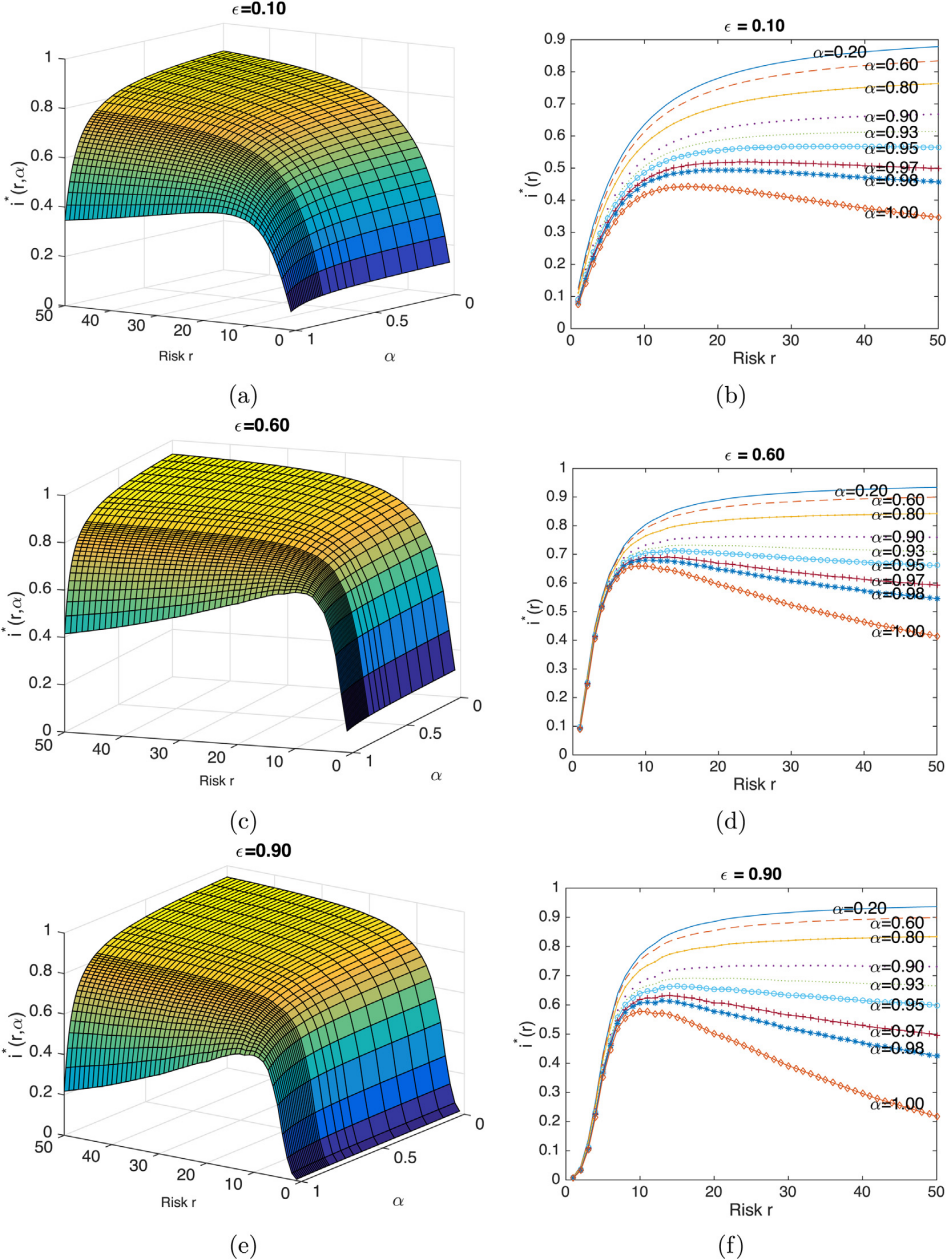
Surface plots of fraction of the population infectious, $i^{\text{*}}\left(r,\alpha \right)$, at steady state versus *r* and *α*, for preference levels (a) ε=0.1, (c) ε=0.6, and (e) ε=0.9. Slices of the 3D surfaces versus *r*, $i^{\text{*}}\left(r\right)$, for different *α* values and preference levels (b) ε=0.1, (d) ε=0.6, and (f) ε=0.9. When α<0.95, the $i^{\text{*}}$ increases with risk *r*. When high-risk people use condoms most of the time, α>0.95, then $i^{\text{*}}$ decreases in the higher-risk groups as a function of *r*.

Fig. 3a – 3f plot the endemic infection distribution as a function of the risk (number of partners): 1) Random mixing (ε=0.1) where 90% of the partners are chosen randomly form the population, 2) Balanced mixing (ε=0.6) where all but 60% of the partners have similar risk behavior, and 3) Highly biased mixing (ε=0.9) where all but 90% of the partners have similar risk behavior. For all values of risk, the fraction of the population infectious at steady state, $i^{\text{*}}$, decreases as condom-use, *α*, increases.

In Fig. 3a, 3c, and 3e, the *α* axis is between α=1 where the high-risk population uses condoms all the time, to α=0 where condoms are never used. The fitted value α=0.69 agrees with Beadnell et al., (2005) studies. For low condom-use (small values of *α*), $i^{\text{*}}$ increases with *r* indicating that a higher percentage of the high-risk people are infectious than the low-risk people. For most values of condom-use, α<0.95, having more partners (increase one's risk *r*), increases the likelihood of being infectious.

However, when the high-risk people use condoms most of the time, α≥0.95, while the lower-risk population only uses condoms occasionally, this trend is reversed. This effect is strongest when the mixing is highly biased (ε=0.9) i.e when most of a person's partners have very similar risk. We note that although this is mathematically consistent with our model, it is in an unrealistic parameter range for the population.

**Fig. 4:**
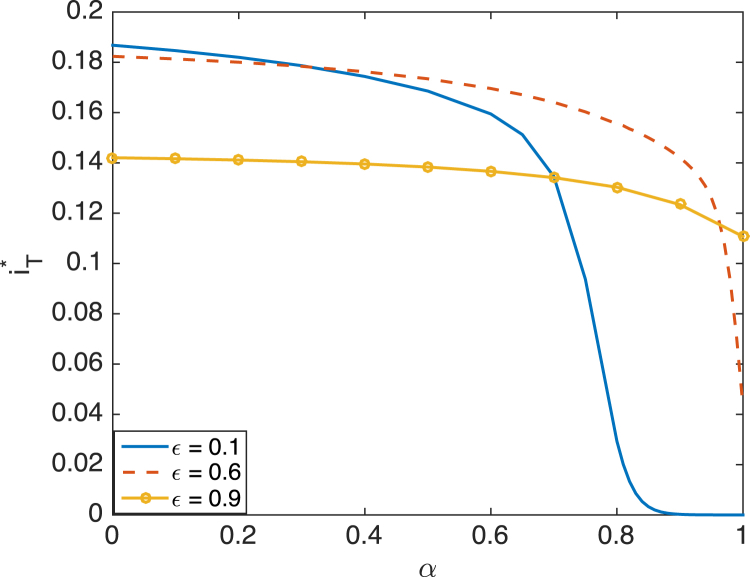
Total fraction of the population that is infectious $i_{T}^{\text{*}}=\int I^{\text{*}}\left(r\right)dr/\int N\left(r\right)dr$ decreases as condom-use *α* increases for random mixing ε=0.1, combined mixing ε=0.6, and highly biased mixing ε=0.9 in partnership selection. Note that when people tend to pick partners randomly, ε=0.1, and the population uses condoms most of the time, α>0.8, then condom-use is an effective way to control the epidemic.

The effectiveness of condom-use in reducing the prevalence is shown in Fig. 4 through the changes in fraction of the total population infectious as a function of *α*, $\left(i_{T}^{\text{*}}=\int I^{\text{*}}\left(r\right)dr/\int N\left(r\right)dr\right)$ for different preference levels *ε*. We found a threshold for *α* to drops the epidemic down, and this threshold increases as mixing level *ε* increases. For example when level of mixing is ε=0.1 (Random mixing), to drop the prevalence drastically, *α* needs to be around 70%, however, for when ε=0.6 (Combined mixing) this threshold is α=0.9, but for ε=0.9 (Highly biased mixing) threshold disappears which means condom-use by high-risk individuals does not have impact on controlling the prevalence. The reason is when people mix more randomly, then high-risk people have many partners with different risks, therefore, using more condom by them save this many partners with different risks, however, when mixing tends to be more biased, ε=0.9, most of the partners of high-risk people are themselves high-risk, which this case this group does not take heavy toll on the prevalence, no mater what fraction of their contact they use condom.

### 4.3 Condom-use scenarios

We compare three condom-use scenarios to quantify their impact on reducing the prevalence of the STI at the endemic equilibrium.

• **NCU** = **no condom-use**: The unrealistic case where condoms are never used is included as a reference case.
• **SCU** = **some condom users**: The population is divided into condom users and non-users where in each risk group, $\hat{c}$ fraction of N(r) of the people use condom all the time, while $\left(1-\hat{c}\right)$ fraction of them never use a condom.
• **FCU** = **fraction condom users**: Everyone uses a condom with probability $\bar{c}$ in each contact. That is, $c\left(r\right)=\bar{c}$ is constant
• **RCU** = **risk-based condom-use**: The condom-use is a function of risk based on the function c(r,α) in equation (2.10) and the scaling parameter *α* is chosen so that the average condom-use is $\langle c\left(r,\alpha \right)\rangle =\bar{c}\text{.}$

**Fig. 5:**
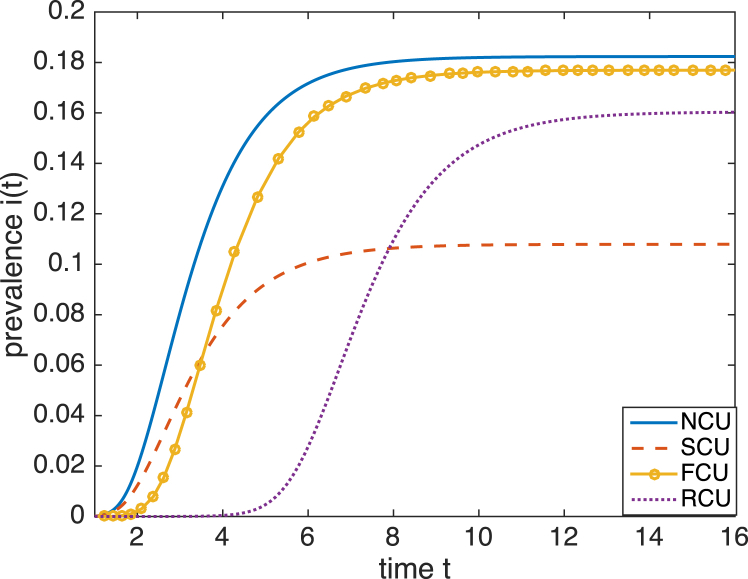
The prevalence of STI as a function of time for different Scenarios: NCU = no condom-use, SCU = sometime condom user, FCU = fraction condom user, RCU = risk-based condom-use where $\hat{c}=\bar{c}=0.37$ and α=0.74 and ε=0.8.

To study the influence of different scenarios on the total prevalence, we recorded prevalence at time *t* for each of them. In Fig. 5, the prevalence for all scenarios are shown as a function of time *t* for $\hat{c}=\bar{c}=0.37$. When condoms are never used (NCU), the prevalence tends to $i\left(t\right)\to i_{T}^{\text{*}}=0.18$. The prevalence is reduced the most for SCU when $\hat{c}=0.37\text{\%}$ of population uses condoms all times. In this case, we observe a reduction of 7% of prevalence at steady state. The reason is that condom-use comes by contact, and when $\hat{c}=37\text{\%}$ of population use condoms in all their contacts, then 37% of population are rarely infectious. On the other hand, for Scenario FCU, i.e when all people use condom $\bar{c}=0.37$ of the contacts, the reduction of prevalence is very weak, almost 0.5%, and this is because the model is applied for highly infectious STIs, that is the chance of catching or transmitting the infection by one contact is high, therefore, even if all people use condom partially, there is a high chance of infection transmission in the contacts which condom is not used.

In the Scenario RCU, i.e using Equation (2.10), when $\bar{c}=0.37$ which results α=0.75, the prevalence at steady state reduces by 2%. In this Scenario, people on average use a condom in 37% of their contacts, however, high-risk people are more likely to us a condom. As we observe, for this Scenario, the growth of infection is slower than the other Scenarios and it takes more time (around 10 years) to reach steady state. This is because, high-risk individuals, who are mostly responsible of spreading infection, use condom more and then transmit or catch infection less than the other Scenarios, therefore, it takes time for them to transmit or catch infection.

## 5 Discussion and conclusions

We developed a simple continuous-risk SIS transmission model for the spread of highly infectious STIs with biased mixing partnership selection to investigate the impact that condoms can have in controlling diseases spread. The model incorporates functions describing mixing patterns as well as condom-use by individuals based on their risk. The mixing between people of different risks was modeled as a combination of random mixing and biased mixing, where people may prefer partners of similar risk (Hyman & Stanley, 1989). Our model includes the observed correlation between condom-use and the number of partners among adolescents and young adults (Beadnell et al., 2005, Kabiru and Orpinas, 2009, Lescano et al., 2006) where people with higher number of partners are more likely to use condoms. We fitted an increasing function of risk for condom-use to the information provided in Beadnell et al., (2005). We assumed that people with more partners (higher risk *r*) were less picky about the risk of their partners than people with fewer partners. We modeled this increased acceptance of the risk of the partners by increasing the standard deviation of risk of the partners as the square root of risk. Other factors, such as alcohol or drug use (Desiderato & Crawford, 1995), that can affect sexual activity and condom-use are not directly included in the model.

The model investigates the role of the risk-structure and importance of homophily in the mixing between people with different risk on the spread of the epidemic. The parameters for the model are chosen to be appropriate for the spread of chlamydia in a cohort of young adults (15−25 years old) in the New Orleans metropolitan area. We do not include sexual structure or aging in the model. These limitations restrict the applicability of the model to situations where the mixing patterns, the transmission rates, and infection prevalence is about the same in men and women. The transmission parameters chosen in our simulations are for highly infectious STIs, such as chlamydia and gonorrhea, and conclusions are only valid for these types of situations. We formulated this simplified model because it is easier to analyze and can provide insight into the dynamics of the more complex models that also account situations where these assumptions do not hold.

The endemic infection equilibrium is more sensitive to the rate that the people with bigger risk *r*, where there are fewer contacts per partnership, use condoms than it is for people with smaller risk *r*, where there are more contacts per partnership. When the probability of infection is high for a single contact, as it is in our simulations, then the number of people an infectious person infects is more correlated to the number of partners that he/she has unprotected sex with, than the number of contacts they have. Our model assumes that people with fewer partners have more contacts per partnership than people with more partners. The risk of infection is high for a single contact where condoms are not used, then even failing to use condoms a few times in a partnership is enough to pass on the infection. That is, the model indicates increasing the fraction of times that people with many partners use condoms could be an effective strategy in mitigating an STI.

The current model does not distinguish between men and women. In heterosexual populations, this approximation is only appropriate when the mixing between men and women is symmetric and the infection prevalence is approximately the same in both men and women. We are extending the model to a heterosexual mixing model, similar to our previous model where we only included two risk groups (Azizi et al., 2016). The heterosexual model can be used to more closely match partnership studies that show, on average, a sexually active man will have more partners than a sexually active woman in the adolescents and young adult population. It can also be used to study the relative effectiveness of increasing the screening for men, women, or both sexes for STIs when there are limited resources.

The simulations quantified the rate that highly infectious STIs like chlamydia spread through a population based on different distributions of condom-use as a function of the population risk. We estimated the impact of condom-use by higher risk individuals on the distribution of endemic equilibrium. We found that for almost all amount of condom-use, having more partner increases the likelihood of being infectious, the infection prevalence is greatest in the higher risk populations and it is always a good mitigation strategy to increase condom-use in these populations to mitigate an epidemic. This effect is stronger when people select most of their partners preferentially.

We also observed that the total prevalence does not drop drastically unless the mixing tends to be more random and high-risk individuals use condom in at least 70% of their contacts. However, when the mixing tends more toward biased mixing, prevalence at steady state looses its sensitivity to condom-use. Our simulations, also, demonstrate that when level of biased mixing is low, then it is also an effective mitigation strategy to increase condom-use in the lower risk populations, as shown in Fig. 4.

We derived the basic reproduction number $R_{0}$ using the next generation approach (Diekmann et al., 1990) and used simulations to show the early growth of the epidemic depends on mixing pattern and condom-use. For very biased mixing, when people pick their partners to have similar risk, then condoms are effective approaches to mitigate the spread of the STI. However, when the population mixed more randomly, then condom-use is less effective in controlling the epidemic.

We created a model with a symmetry in the parameters and behaviors for men and women which is appropriate for some STIs like chlamydia, syphilis, and gonorrhea and also it is appropriate for homosexual STIs. The model has a flexibility to be extended for heterosexual population with different parameter values and behaviors by men and women, two-sex bipartite model (Hyman & Stanley, 1994). The model in this paper is not appropriate for spread of HIV/AIDS where the prevalence and transmission rates between men and women are low and asymmetrical. Also, the prevalence of HIV/AIDS in men and women is a strong function of age in the 15−25 year-old age group and should be accounted for in a heterosexual HIV/AIDS model.

We recognize that a more realistic approach is needed for guiding public health policy. This realistic model would track behavior change and mixing based on a person's age. For example, when an individual is infected and treated, then they are more likely to change their behavior to prevent being infected again. Behavior change is an important assumption which could be added in this model by including risk-based partial derivative terms. This extension would make the model significantly more complex and would not be as good as using an agent-based model that can follow the infection status of each individual.

The analysis and simulations of our continuous-risk model has led us in creating a more appropriate model for studying the impact of screening, contact tracing, partner treatment, condom-use, and behavior change in controlling the spread of STIs (Azizi & Hyman, 2017). We are formulating a stochastic (Monte Carlo - Markov Chain) agent-based bipartite disease-transmission network-model where the men and women are the network nodes and sexual contact are represented by edges between the nodes. The network captures the distributions for number of partners that men and women have, and the correlations between the number of partners that a person has and the number of partners their partners have. These partnership distributions, and the transmission parameters, are based on survey data for the 15−25 year-old African American community in New Orleans.

Unlike the continuous-risk model, the network model can track an individual's behavior change, such as condom-use after being treated for infection, the affect of aging on number of partners a person has, or the differences in condom-use between primary and casual partners. Unfortunately, the complexity of the network model makes the mathematical analysis far more difficult than the continuous-risk model described in this paper. Our future research will be guided by combining the mathematical analysis of the simplified model, described in this paper, with simulations of the more realistic agent-based model to help guide in the public health efforts mitigating the spread of chlamydia as a highly infectious STI.
